# Transcriptomic, proteomic, metabolomic, and functional genomic approaches of *Brassica napus* L. during salt stress

**DOI:** 10.1371/journal.pone.0262587

**Published:** 2022-03-10

**Authors:** Jiabin Shu, Xiao Ma, Hua Ma, Qiurong Huang, Ye Zhang, Mei Guan, Chunyun Guan

**Affiliations:** 1 The Oilseed Crop Research Institute, National Oilseed Crop Improvement Center (Hunan), Hunan Agricultural University, Hunan, China; 2 Quzhou Academy of Agricultural and Forestry Sciences, Quzhou, Zhejiang, China; Southwest University, CHINA

## Abstract

Environmental abiotic stresses limit plant growth, development, and reproduction. This study aims to reveal the response of *Brassica napus* to salt stress. Here, transcriptomics, metabolomics, and proteomics analysis were performed on 15 *Brassica napus* leave samples treated with salt at different times. Through functional enrichment analyzing the differentially expressed genes (DEGs), differential metabolites (DMs) and differentially expressed proteins (DEPs), the key factors that dominate *Brassica napus* response to salt stress were identified. The results showed that the two key hormones responding to salt stress were Abscisic acid (ABA) and jasmonic acid (JA). Salt stress for 24h is an important milestone. *Brassica napus* adjusted multiple pathways at 24h to avoid over-response to salt stress and cause energy consumption. The increased expression in *BnPP2C* is tangible evidence. In response to salt stress, JA and ABA work together to reduce the damage caused by salt stress in *Brassica napus*. The increased expression of all BnJAZs after salt stress highlighted the function of JA that cannot be ignored responding to salt stress. In addition, some metabolites, such as N-acetyl-5-hydroxytryptamine, L-Cysteine and L-(+)-Arginine, play a critical role in maintaining the balance of ROS. Proteins like catalase-3, cysteine desulfurase, HSP90 and P450_97A3 were the most critical differential proteins in response to salt stress. These findings of this study provide data support for *Brassica napus* breeding.

## Introduction

Abiotic stress seriously restricts plant growth, development, reproduction, and life [1–3]. When plants are subjected to salt stress, Na^+^ and Cl^−^accumulation in the cytoplasm lead to cytotoxicity by reactive oxygen species (ROS), which causes protein and lipid degradation and cell disruption [4, 5]. In the adaptation process, salt stress regulatory genes are induced, leading to changes in secondary metabolites and protein levels that enable adaptation to the salinity conditions [6]. The molecular mechanisms involved in salt stress have been investigated, and many genes and proteins have been identified to play roles in enhancing tolerance to salt stress in plants [7]. For example, jasmonates are known to take part in various physiological processes. Exogenous application of jasmonates has proved effective in improving plant stress tolerance, such as salinity stress, drought stress, and low/high-temperature stress [8]. Previous studies reported that signal transduction pathways, including abscisic acid (ABA) signal transduction, WRKY related pathways, etc., are activated. Many defense response-related genes are induced, ultimately confer salt tolerance on the surface the plant [9]. Munns and Jamil summarized the defense response-related proteins: proteins involved in signal transduction, transcription factors (TFs), proteins involved in osmolyte synthesis, antioxidant proteins, and other genes induced by salt treatments, such as HSP (heat shock protein), P450, *etc*. [10, 11].

*Brassica napus* is widely cultivated worldwide and is an important oil crop, mainly used for human consumption and animal feed [12]. Although the production of *Brassica napus* is increasing, environmental stresses are critical limiting factors. Like other important crops, salt stress reduces *Brassica napus* yield and quality. Several articles have been reviewed the morphological, physiological, and biochemical response of *Brassica napus* to salt stress [13, 14]. When *Brassica napus* is subjected to salt stress, cells mobilize multiple components in response to stress. Some studies have demonstrated the stress response mechanism of salt stress in *Brassica napus* at the gene expression level [15, 16], but this is far from enough. Metabolites such as sugars, organic acids, polyols, amino acids, amides, imino acids, ectoine, proteins, and quaternary ammonium compounds have been proven to play essential roles in the tolerance of toxicity ions under salt stress [13]. Meanwhile, genes also actively respond to salt stress signals, and the expression levels of some genes change dramatically in a short time to synthesize proteins needed to respond to stress. Some genes are suppressed under salt stress. The development of biotechnological methods dedicated to plant transcriptomics analysis provides a valuable opportunity to reveal the basis of plant response to salt stress at the molecular level [17]. With the continuous advancement of technology, we can obtain the transcriptome expression profile in plant cells and use liquid chromatography/mass spectrometry (LC/MS) technology to identify and analyze metabolites and proteins in the plant [18]. Integrative omics approaches within large-scale experiments, including genomics, transcriptomics, ionomics, proteomics, and metabolomics, can help decipher the interplay of cellular functions at different levels [19]. Based on this, we can reveal a series of response mechanisms triggered by cells in response to salt stress. Transcriptomics, combined with metabolomics and proteomics, provides a major tool for the characterization of postgenomic processes [20]. The combination of transcriptomics, metabolomics, and proteomics has been applied to reveal stress response mechanisms in various plants [21, 22]. However, there is no similar multi-omics study on the response of *Brassica napus* to salt stress.

Here, we carried out transcriptomics, metabolomics, and proteomics analysis of 15 *Brassica napus* leave samples treated with salt at different times to reveal salt stress response mechanisms. Through analyzing the differentially expressed genes (DEGs), differential metabolites (DMs), and differentially expressed proteins (DEPs), we obtained the key factors that dominate *Brassica napus* response to salt stress. Furthermore, these findings provide data support for the breeding of *Brassica napus*.

## Materials and methods

### Plant materials

The *Brassica_napus* cv. *Yanyouza No*.*3* was used as the plant material. The seeds were obtained from the Germplasm Resource Bank in Jiangsu Academy of Agricultural Sciences. The experiment was performed in an Intelligent Artificial Climate Box Control System with 25°C, 16h, and 7000 Lux light intensity, 50% humidity in the daytime, 20°C, 8h, and 65% humidity at night.

### Preparing and sowing of seeds

*Brassica_napus* seeds were surface sterilized in mercury dichloride solution (1:1000) for 8 min, rinsed with distilled water, and then transferred to Petri dishes for germination. After three days, germinated seeds (300 grains) were cultivated in a 250 ml plant cultivation pot, which was divided into upper and lower layers. The upper layer was covered with sterilized quartz sand, and the lower layer was filled with Hoagland culture medium. The Hoagland’s nutrient solution consisted of 5 mM Ca (NO_3_)_2_, 5 mM KNO_3_, 1 mM KH_2_PO_4_, 50 M H_3_BO_3_, 1 mM MgSO_4_, 4.5 M MnCl_2_, 3.8 M ZnSO_4_, 0.3 M CuSO_4_, 0.1 mM (NH_4_)_6_ Mo_7_O_24_ and 10 M Fe-EDTA at a pH of 5.5. The culture medium was replaced every five days and the tray was cleaned every ten days. Seedlings with similar heights were selected for salinity treatment at the five-leaves stage.

### Experimental design and salt treatment

A total of 60 *Brassica napus* seedlings with a similar physiological state were allocated to experimental pots. A total of 5 groups were designed in this study, namely CK (the control group), T3, T6, T12 and T24 (plant materials are randomly assigned to each group, each with 12 seedlings). For group CK, the experimental pots were filled with Hoagland’s nutrient solution (No NaCl added). For salt treatment groups, NaCl-containing Hoagland’s nutrient solution (final concentration, 240 mM) was used. The seedlings in T3, T6, T12 and T24 were treated with NaCl for 3h, 6h, 12h and 24h, respectively. After salt treatment, 9 individuals were randomly selected from the 12 seedlings in each group and the third true-leaf of each individual was collected (washing the surface of leaves samples with enzyme-free water three times before collection). Every three leaves were pooled as a biological repeat sample of each group, and a total of 15 samples were collected (five groups in total, with three replicates in each). Then, each biological repeat sample was divided into three parts, which were used for transcriptome, metabolomic, and proteome analysis, respectively.

### Physiological indexes measurement

The flag leaf samples were sampled and pretreated with 0.05mol/L phosphate buffer (pH7.8). They were then ground into slurry under low-temperature conditions. Total superoxide dismutase (SOD) activity and malondialdehyde (MDA) levels were measured using SOD and MDA assay kits (Nanjing Jiancheng Bioengineering Inc., Nanjing, China) in strict accordance with kit requirements, which referred to the method reported by Zhao et al [23]. Each sample’s proline (Pro) content was quantified by ninhydrin labeling using a plant proline assay kit (Nanjing Jiancheng Bioengineering Inc., Nanjing, China). The total Pro content in the leaf sample was expressed as microgram per gram of fresh leaf weight (μg/g). All experiments were carried out three times with triplicates in each experiment.

### RNA extraction and transcriptome analysis

Three biological replicates of each group were analyzed. Frozen leaves were powdered in liquid nitrogen. Total RNA was isolated using a QIAzol lysisreagent (Qiagen, Hilden, Germany) and purified by an RNeasy Plant Mini Kit (Qiagen, Hilden, Germany) following the manufacturer’s protocol. Approximately 3μg of total RNA from each sample was subjected to the RiboMinus Eukaryote Kit (Qiagen, Hilden, Germany) to remove ribosomal RNA prior to the construction of the RNA-seq libraries. RNA-seq libraries were prepared using an RNA-seq Library Prep Kit for Illumina (Vazyme, Nanjing, China). The libraries were sequenced on an Illumina HiSeq 4000 platform using a PE150-bp read module. The raw sequencing reads were filtered and trimmed using the default parameters of FastQC (version 0.11.5). The filtered clean data were then assembled and compared to the reference genome of *Brassica_napus* (https://www.ncbi.nlm.nih.gov/assembly/GCF_000686985.2/) using hisat2 (http://ccb.jhu.edu/software/hisat2) with the default parameters. The value of FPKM of reads was calculated using Cufflinks (version 2.2.1, http://cole-trapnell-lab.github.io/cufflinks/) and differentially expressed genes (DEGs) were identified using DESeq2 (http://bioconductor.org/packages/release/bioc/html/DESeq.html) with the criteria of *q* value (adjusted *p* value, benjamini-hochberg method) <0.05 and |log2(Fold Change, FC)|≥ 2. The genes with the false discovery rate (FDR) parameter below 0.05 and absolute fold change≥2 were considered DEGs. Correlation analysis was performed by R (V3.3.2, https://www.r-project.org/). The correlation of two parallel experiments provides the evaluation of the reliability of experimental results as well as operational stability. The correlation coefficient between three replicas was calculated to evaluate repeatability between samples. Principal component analysis (PCA) was performed with R package gmodels (http://www.rproject.org/) to convert hundreds of thousands of correlated variables (gene expression) into a set of values of linearly uncorrelated variables called principal components. Moreover, we investigated the functions of the DEGs using Gene Oncology (GO) and Kyoto Encyclopedia of Genes and Genomes (KEGG) pathway analysis using blast2go software (v.4.1.995, https://www.blast2go.com/). Significant GO terms and KEGG pathways were identified with the criterion of q value <0.05.

### LC-ESI-MS/MS analyzing and data p rocessing

Leave samples were placed in a forced-air oven (55°C) and then pulverized in a Wiley mill. The powdered samples were extracted (1g) twice in 20 mL of 70% cold methanol using an ultrasonicator for 60min at 4°C each time for each sample. After centrifuging at 12000 rpm/min for 15 minutes at 4°C, the supernatant was transferred to a new tube and stored at -80°C. The compounds extracted were analyzed using an LC-ESI-MS/MS system (UPLC, Shim-pack UFLC SHIMADZU CBM30A) and MS/MS (Applied Biosystems 6500 QTRAP) under both the ESI negative and positive mode.

The Analyst 1.6.1 software recorded the data. Peak data filtering was fixed at a standard of signal/noise (s/n) > 10. Metabolites were then identified by searching our internal database and public databases, including MassBank (http://www.massbank.jp/), KNApSAcK (http://www.knapsackfamily.com/KNApSAcK/), HMDB (http://www.hmdb.ca/), MoTo DB (http://www.ab.wur.nl/moto/), and METLIN (https://metlin.scripps.edu/). For a preliminary visualization of differences between different groups of samples, the unsupervised dimensionality reduction method principal component analysis (PCA) was applied in all samples using R software (V3.2.2, http://www.r-project.org/). All samples were tested to visualize the metabolic alterations by principal component analysis and (orthogonal) partial least-squares-discriminant analysis OPLS-DA. The models were developed using SIMCA-P software (v. 15.0, Umetrics, Sweden) following Mandrone’s report [24]. To rank the metabolites that distinguished between two groups, variable importance in projection (VIP) score of (O)PLS model was calculated. The threshold of VIP was set to 1. In addition, the T-test was used as a univariate analysis for screening DMs. Finally, the DMs were mapped to KEGG metabolic pathways for enrichment analysis.

### HPLC-MS/MS analyzing and protein data processing

Samples were ground to powder in liquid nitrogen, then dissolved in 2mL lysis buffer containing 8M urea, 2% SDS, and 1× Protease Inhibitor Cocktail (Roche, Switzerland), followed by sonication on ice for 30min and centrifugation at 13 000 rpm for 30min at 4°C. The supernatant was treated with ice-cold acetone at -20°C overnight. Afterwards, the precipitations were washed thrice with acetone and then re-dissolved in 8M Urea by sonication on ice. Protein quality was examined with SDS-PAGE. The peptides were dissolved in 30 μL solvent A (A: 0.1% formic acid in water) and analyzed by online nanospray LC-MS/MS on an Orbitrap Fusion Lumos coupled to EASY-nLC 1200 system (Thermo, Waltham, MA, USA) [25]. The analysis parameters used in this study were set as follows: for MS, Scan range (m/z) = 350–1200, resolution = 120,000, AGC target = 1e6, maximum injection time = 50 ms; for HCD-MS/MS, resolution = 30,000, AGC target = 1e6, collision energy = 32, stepped CE = 5%; for DIA (Data Independent Acquisition), variable isolation window was used with each window overlapped 1 m/z, and the window number was 60. The raw MS/MS data was downloaded and processed using Spectronaut X (Biognosys AG, Switzerland) with default parameters. According to GO, KEGG, and COG/KOG databases, peptides were annotated. Proteins with a false discovery rate (FDR) <0.01 were retained and used for the identification of differentially expressed proteins (DEPs). GO and KEGG enrichment analysis results were carried out to analyze the functional classification of DEPs.

### qRT-PCR

The quantitative real-time PCR was performed as described previously [26]. Briefly, 500 ng mRNA was used for cDNA synthesis using Vazyme R223-01 HiScript II Q RT SuperMix for qPCR (Vazyme, Nanjing, China). Quantitative PCR was performed in technical triplicate 20 μl reactions using 2× ChamQ SYBR qPCR Master Mix (Vazyme, Nanjing, China) run on an ABI Step One Plus qPCR instrument using gene-specific primers (**S1 Table**). Beta-actin (B-ACT), the commonly used internal reference gene [27], was used as a housekeeping gene to normalize gene expression. Data were analyzed with the 2^-ΔΔCt^ method.

## Results

### Effects of salt stress on physiological indexes

**Fig 1:**
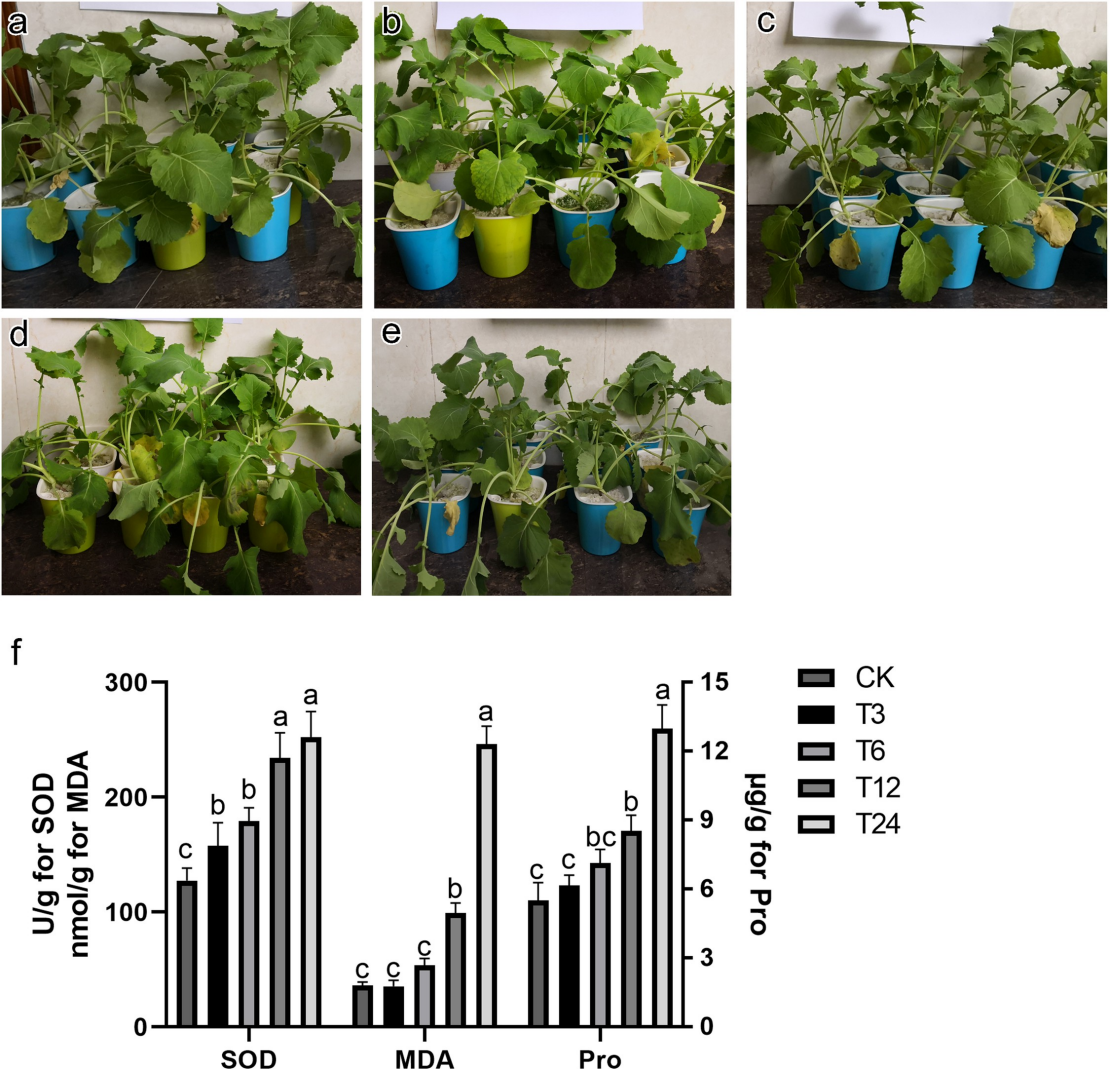
Effects of salt stress on phenotype and physiological indexes. (a) to (e) shows the phenotype of Brassica napus seedlings treated with salt stress. (a) to (e) represents CK, T3, T6, T12 and T24 groups, respectively. (f). The image shows the total superoxide dismutase activity (SOD, left-hand ordinate), malondialdehyde levels (MDA, left-hand ordinate) and the amount of proline (Pro, right-hand ordinate) in different groups. Identical letters indicate the absence of a significant difference (*p*>0.05) and different letters indicate a significant difference (*p*<0.05).

After salt treatment, the plant leaves did not show obvious signs in the early stage, but showed slight wilting at 24 hours (**Fig 1A**). Physiological indexes of *Brassica napus* after salt stress results showed that the SOD, MDA and Pro gradually increased with salt stress time (**Fig 1B**). The SOD activity in T24 showed no significant difference with T12, but they were significantly higher than that in T6 and T3, which were significantly higher than that of CK. MDA activity had the highest value at 24h, which was significantly higher than the other groups. There was no significant difference found among the T6, T3, and CK groups, indicating that MDA activity was more inclined to respond to the salt stress with a longer duration (over 12h). Similarly, the content of Pro in T24 group, the highest of all groups, was significantly higher than that in T12, T6, T3 and CK. T12 was significantly higher than that in T3 and CK.

### Differential transcriptome analysis

A total number of 802 million short reads (82.21 Gb) were generated from the 15 libraries with an average of 53.47 million reads per sample (**S2 Table**). All the reads were mapped to the *Brassica napus* reference genome, with an average alignment rate of 91.29%. The PCA results showed that samples in T12 and T24 were separated from the other groups. The clustered samples in CK, T3, and T6 were relatively close (**S1A Fig**). Sample-to-sample correlation analysis results showed a clear distinction between T12 and T24 (**S1B Fig**). Totally, 374, 2324, 8500, and 12542 DEGs were identified in comparing CK *vs*. T3, CK *vs*. T6, CK *vs*. T12, and CK *vs*. T24, respectively (**S1C Fig**). The number of DEGs increased with the duration of salt treatment. The Venn analysis results showed that only 68 genes are commonly expressed in all the comparisons (**S1D Fig**).

**Fig 2:**
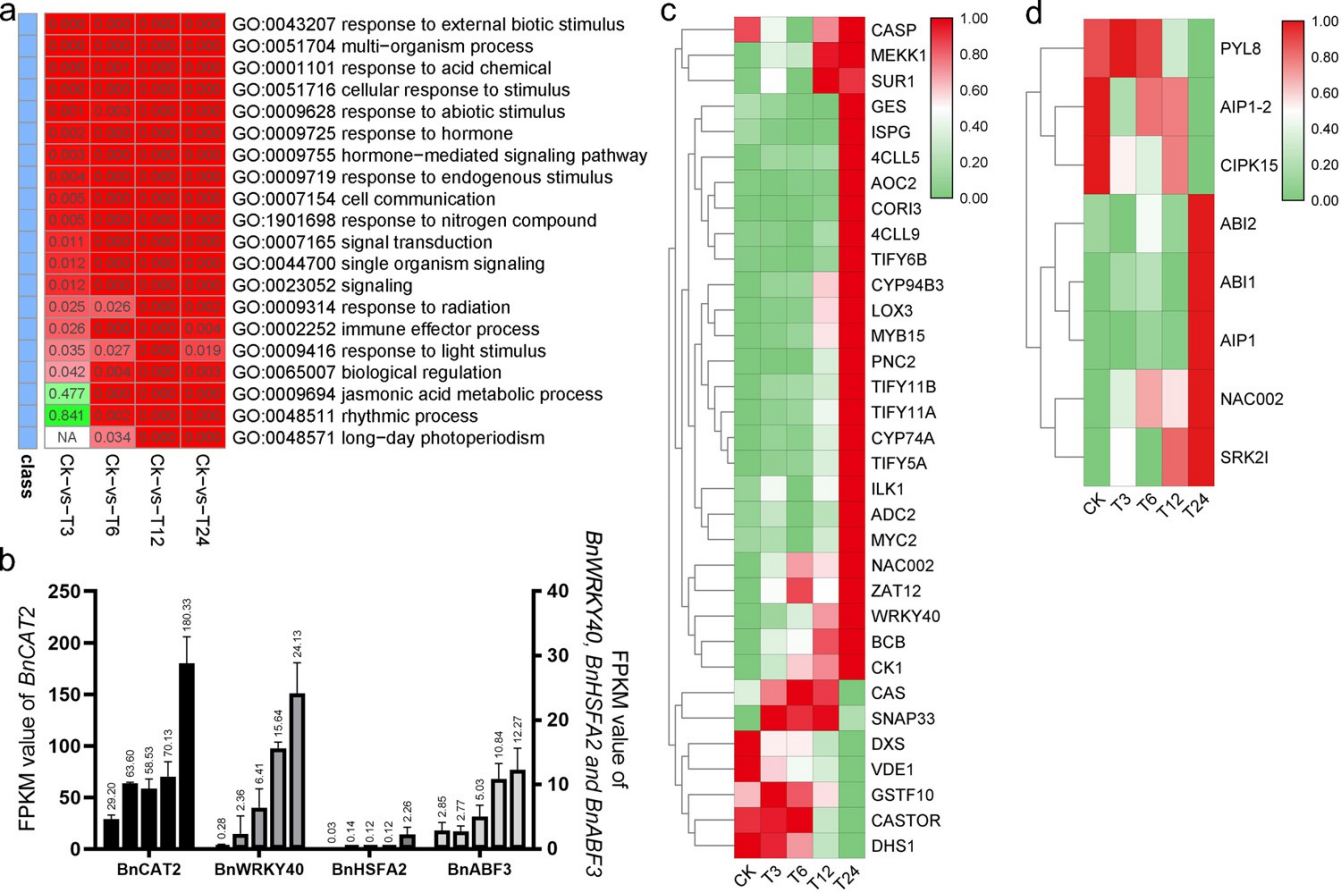
Gene Ontology (GO) clustering results and expression of key regulators. (a). Heatmap of GO Biological process terms in different comparisons. Red cell means *p*<0.05, the redder the color, the lower the *p*-value. green cell means *p*>0.05, the greener the color, the higher the *p*-value. The figures in the cells showed the *p*-value. (b) represents the expression level of four transcription factors. The number on the bar chart represents the average. (c) and (d) represent the expression of DEGs in jasmonic acid and abscisic acid signaling pathway, respectively. Red cell means *p*<0.05, the redder the color, the lower the *p*-value. green cell means *p*>0.05, the greener the color, the higher the *p*-value.

**Fig 3:**
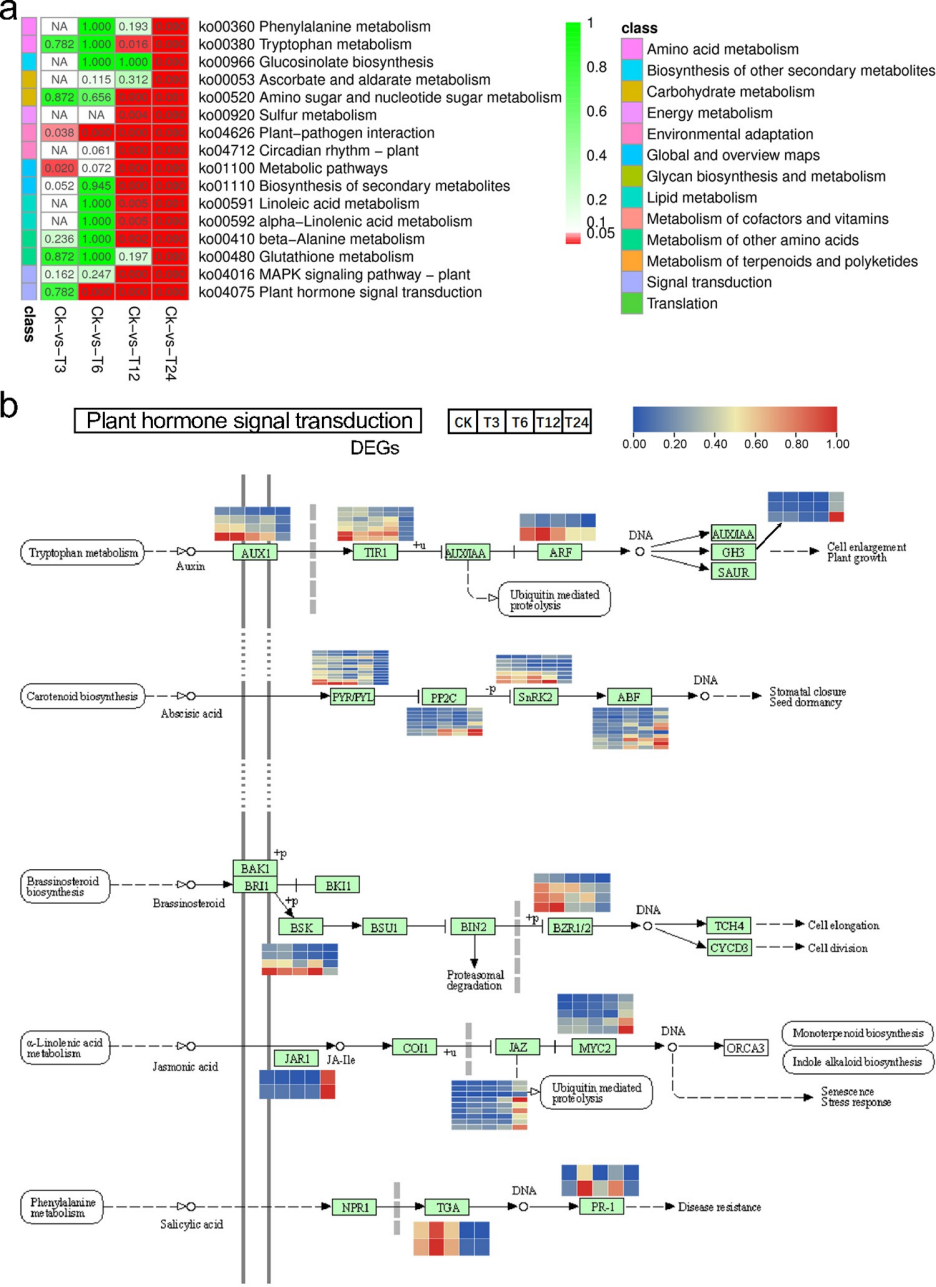
Kyoto Encyclopedia of Genes and Genomes (KEGG) pathway analysis results and expression of key regulators. (a). Heatmap of KEGG pathways in different comparisons. Red cell means *p*<0.05, the redder the color, the lower the *p*-value. green cell means *p*>0.05, the greener the color, the higher the *p*-value. The figures in the cells respects the *p*-value. (b) shows the expression profiles of DEGs in plant hormone signal transduction pathway. Blue and red cell respect a decrease and an increase of a DEG, respectively.

GO enrichment analysis was performed on the DEGs in each comparison. GO terms related to phytohormone responses, including “response to stress” (GO:0006950), “hormone-mediated signaling pathway” (GO:0009755), “response to abiotic stimulus” (GO:0009628), “jasmonic acid metabolic process” (GO:0009694) etc., were significantly enriched in all these four comparisons (**Fig 2A**). A detailed analysis of the members in these mentioned GO terms revealed four transcription factors, namely *BnCAT2*, *BnWRKY40*, *BnHSFA2*, and *BnABF3* were activated in leave by salt stress and reached the peak at 24 hours (**Fig 2B**). In addition, DEGs related to jasmonic acid were also *Bn4CLL5*, *Bn4CLL9*, *BnADC2*, *BnDTX9*, *BnGES*, *BnVSP2*, *BnJAR1*, etc., sustained high expression values through our salt treatment experiment (**Fig 2C**). Meanwhile, the expression of abscisic acid (ABA) related DEGs were also increased by salt stress, such as *BnPYL8*, *BnPUB9*, *BnCIPK15*, *BnABI1*, *BnNAC002*, *BnSRK2I*, etc (**Fig 2D**). Then, we clustered these DEGs with the KEGG database, the results showed that “Plant hormone signal transduction” (ko04075), “Plant-pathogen interaction” (ko04626), “Glutathione metabolism” (ko00480), “MAPK signaling pathway-plant” (ko04016), etc., were significantly enriched (**Fig 3A**). In particular, DEGs in the plant hormone signal transduction pathway deserve our attention. In this pathway map, many DEGs and their gene family members were strongly stimulated by salt stress (**Fig 3B**), including *BnAUX*, *BnARF*, *BnABF*, *BnPYL*, *BnJZR*, *BnJAZ*, etc. Some of them have already been mentioned in the GO enrichment analysis.

### DMs and functional clustering

**Fig 4:**
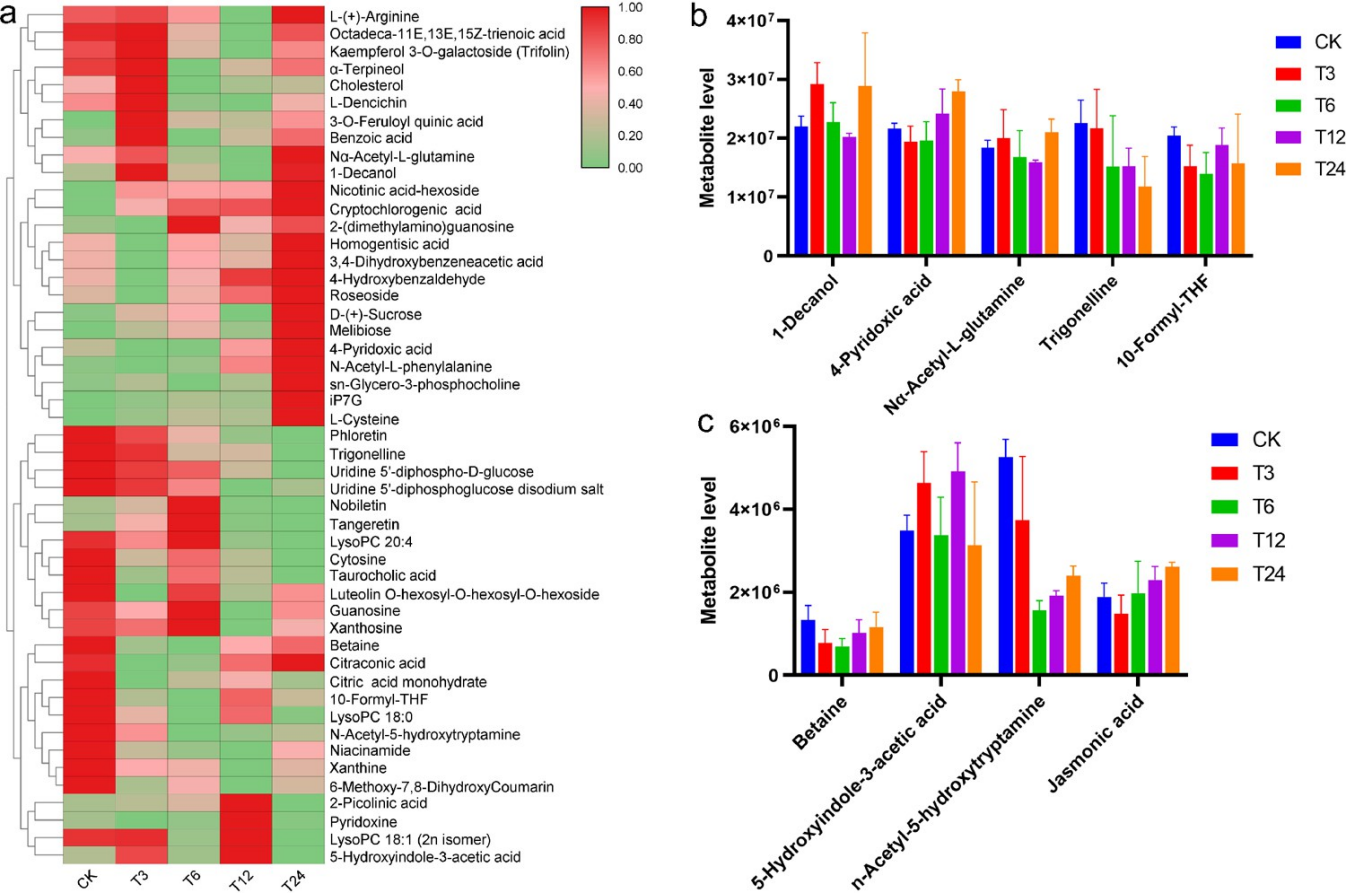
Content of differential metabolites. (a). The levels of differential metabolites. The redder the color, the higher level of differential metabolites. (b). The contents of the five differential metabolites with the highest abundance. (c). The content of key differential metabolites screened according to KEGG enrichment results.

The quality control results of LC-MS raw data can ensure the availability of data. PCA and PLS-DA revealed a clear separation of the salt treatment and control group (**S2A to S2E Fig**). Totally, 49 DMs were identified and the heatmap of the DMs was shown in **Fig 4A**. To uncover the most relevant biological pathways of salt stress, we perform functional annotation and cluster analysis of these 49 DMs. Firstly, the five most abundant DMs were identified, including 1-Decanol, 4-Pyridoxic acid, Nα-Acetyl-L-glutamine, Trigonelline and 10-Formyl-THF, the contents of these metabolites decreased gradually with the extension of salt stress time (**Fig 4B**). Secondly, functional annotation results showed that some metabolites were related to salt stress, such as 10-Formyl-THF, betaine, 5-Hydroxyindole-3-acetic acid (5-HIAA), n-Acetyl-5-hydroxytryptamine and Jasmonic acid (**Fig 4C**). As we know, betaine can maintain plant cell osmotic balance; however, its content decreased first and then increased. Jasmonic acid, a critical signaling molecule in regulating gene expression under salt stresses, was increased with the extension of salt stress time. N-acetyl-5-hydroxytryptamine can affect the levels of a number of ROS metabolites. N-Acetyl-5-hydroxytryptamine levels decreased 0.3-fold, 0.7-fold, 0.6-fold and 0.5-fold in the plants exposed to 3h, 6h, 12h and 24h salt treatment, respectively, compared to the control.

Further, we performed KEGG enrichment analysis on all DMs between T3 vs CK, T6 vs CK, T12 vs CK and T24 vs CK. The results showed that “Metabolic pathways” (ko01100), “Taurine and hypotaurine metabolism” (ko00430), “C5-Branched dibasic acid metabolism” (ko00660), “Biosynthesis of secondary metabolites” (ko01110) et al. were significantly enrichened (**Table 1**). By further analyzing the DMs in these pathways, we found that N-Acetyl-5-hydroxytryptamine, L-Cysteine and L-(+)-Arginine were involved in multiple pathways. The content change of N-Acetyl-5-hydroxytryptamine has been described above. The content of L-Cysteine was increased by salt treatment and peaked at 24h. L-(+)-Arginine reached the lowest level at 12h, but maintained a relatively similar content at other times (**Fig 4C**).

**Table 1:** KEGG enrichment analysis results on all DMs.

Comparison	Pathways	P value	Metabolites
CK vs. T3	C5-Branched dibasic acid metabolism	0.02851912	Citraconic acid
CK vs. T6	One carbon pool by folate	0.02508961	Cholesterol
			Niacinamide
	Metabolic pathways		10-Formyl-THF
	C5-Branched dibasic acid metabolism		Xanthine
		0.03800457	Betaine
		0.04963771	
			Citraconic acid
			N-Acetyl-5-hydroxytryptamine
CK vs. T12	Tryptophan metabolism	0.009858911	Xanthine
			Guanosine
	Caffeine metabolism		2-Picolinic acid
		0.01094605	
		0.0274495	
	Purine metabolism		Xanthosine
			5-Hydroxyindole-3-acetic acid
			N-Acetyl-5-hydroxytryptamine
CK vs. T24	Galactose metabolism	0.01700451	Uridine 5’-diphospho-D-glucose
	Taurine and hypotaurine metabolism		L-Cysteine
			D-(+)-Sucrose
		0.0214922	Taurocholic acid
			Melibiose

Hence, betaine, jasmonic acid, N-acetyl-5-hydroxytryptamine, L-Cysteine and L-(+)-Arginine were identified as the key metabolites involved in rapeseed response to salt stress.

### DEPs identification and functional analysis

**Fig 5:**
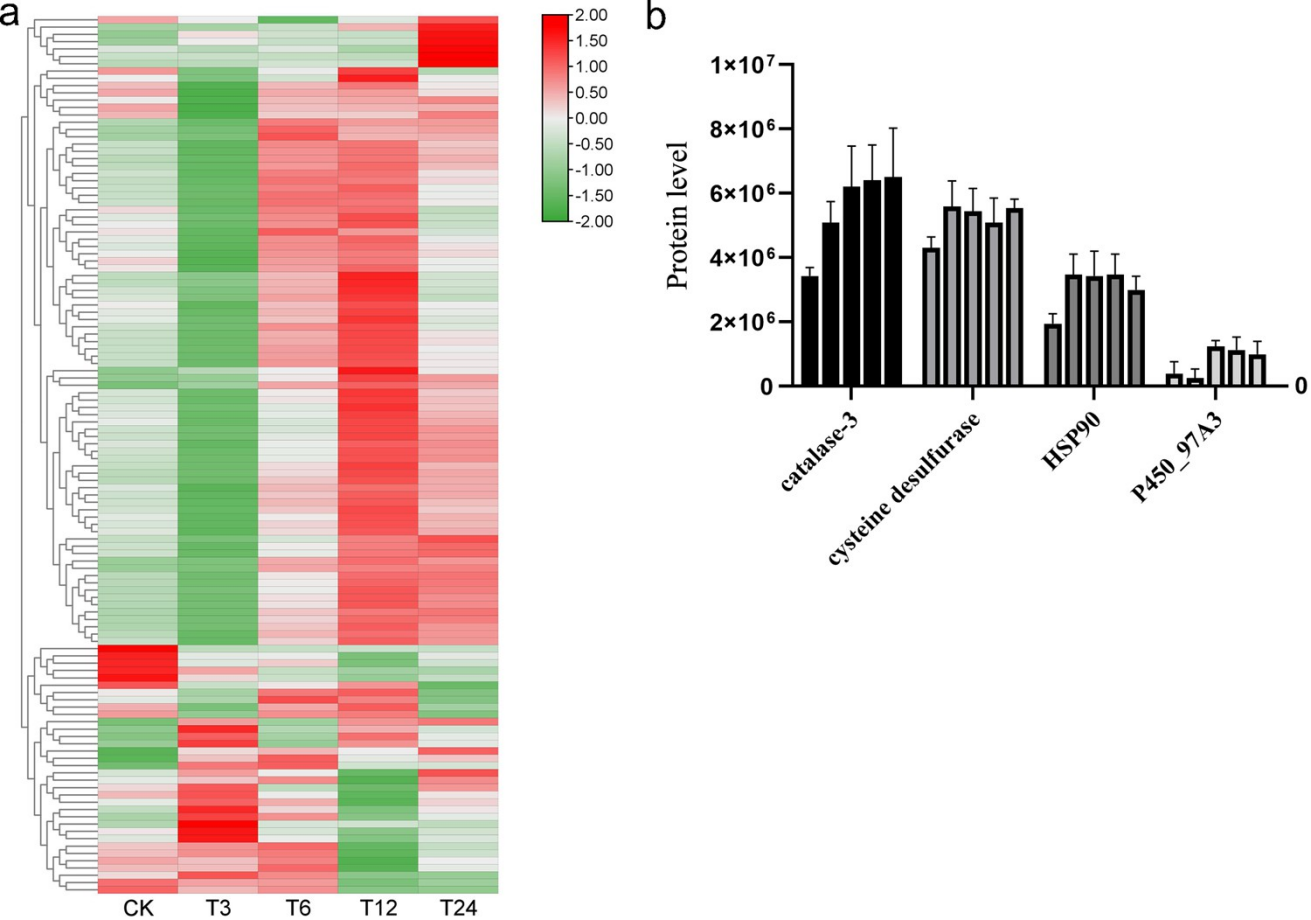
Key Differentially Expressed Proteins (DEPs) levels. (a). The levels of differential expressed proteins. The redder the color, the higher level of differential proteins. (b). The image shows the abundant of catalase-3 (CAT3, left-hand ordinate), cysteine desulfurase, HSP90 protein, and cytochrome P450_97A3 (left-hand ordinate).

In the current study, we performed a DIA quantitative proteomics approach to investigate the potential mechanism of salt stress response. The data showed that there were 124 DEPs were filtered out from the over 10000 proteins that identified in the DIA data. The level of all the DEPs were shown in **Fig 5A**. By annotating these DEPs, we identified several DEPs that related to salt stress, including catalase-3 and HSP90, P450_97A3. These proteins have been reported to be closely related to abiotic stress.

Further, we performed KEGG enrichment analysis base on all DEPs. The results showed that three KEGG pathways were significantly enriched, including “Sulfur metabolism” (ko00920), “Thiamine metabolism” (ko00730) and “Peroxisome” (ko04146) (**Table 2**). According to the results, the salt stress response is mainly related to the cell’s response to peroxisome and sulfur metabolism. After analyzing the content of DEPs in each group, two candidate DEPs, catalase-3 and cysteine desulfurase, were significantly enriched in these pathways. The content of catalase-3 was increased with the increase of salt stress time. Cysteine desulfurase was increased first but then decreased to normal level (**Fig 5B**). In addition, the GO enrichment analysis exhibited that HSP90 and cytochrome P450_97A3 were significantly enriched in “response to stimulus” (GO:0050896) terms (**Table 2**). The content of HSP90 protein and cytochrome P450_97A3 were significantly increased by salt treatment (**Fig 5B**).

**Table 2:** KEGG and GO enrichment analysis results on all DEPs.

Comparison	Pathways	GO terms
CK vs. T3	RNA transport	Nucleotide catabolic process
		Ribonucleotide catabolic process
CK vs. T6	Plant-pathogen interaction	Sulfate assimilation
	Protein processing in endoplasmic reticulum	Photosynthetic electron transport chain
	Sulfur metabolism	Negative regulation of catalytic activity
CK vs. T12	Ribosome	Response to stimulus
	Peroxisome	protein-DNA complex subunit organization
		Gas transport
CK vs. T24	Sulfur relay system	Sulfate assimilation
	Thiamine metabolism	Response to stimulus
	Sulfur metabolism	

Finally, based on the annotation results and cluster analysis of GO and KEGG, we screened out 4 key differentially expressed proteins: catalase-3, cysteine desulfurase, HSP90 and P450_97A3.

### Interactive network analysis

**Fig 6:**
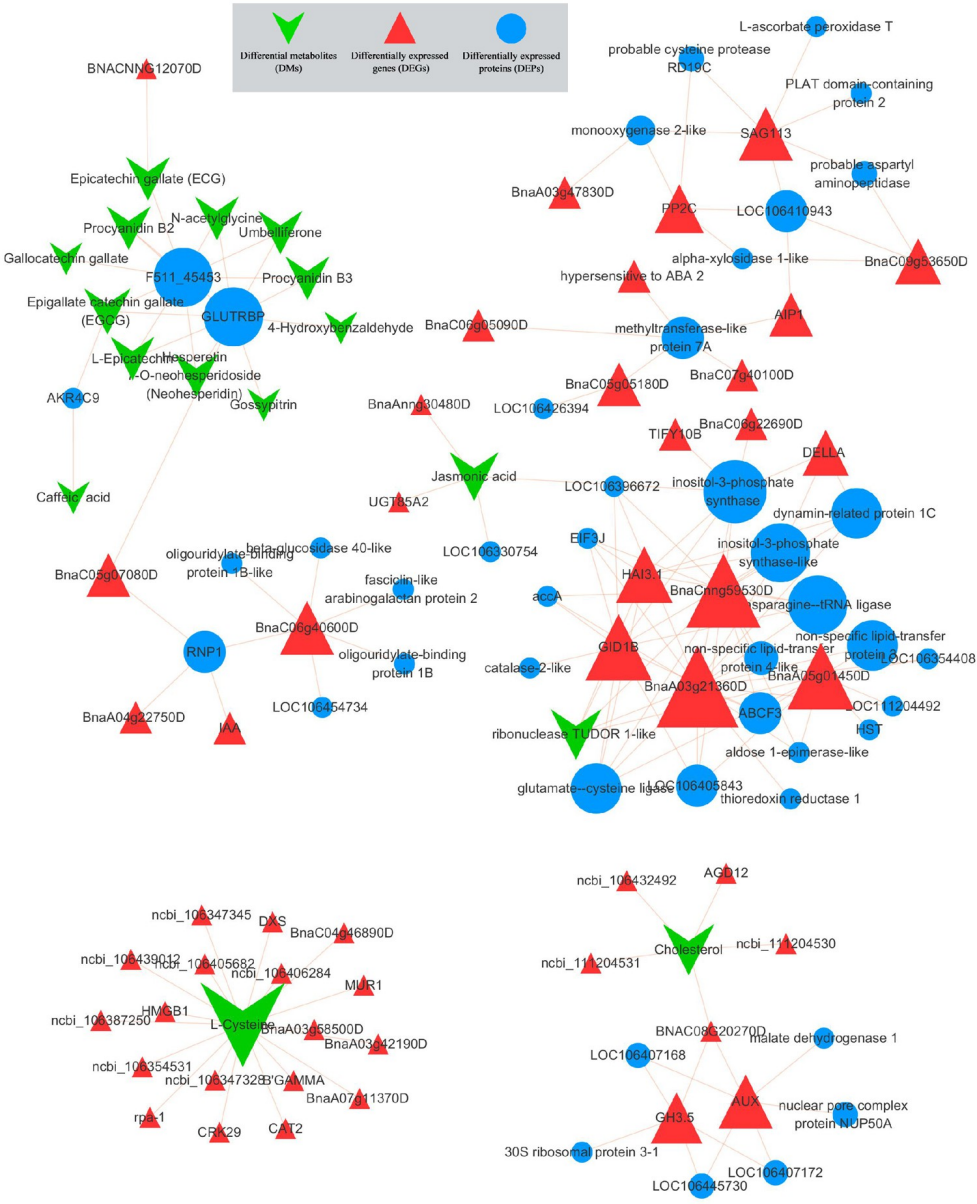
Transcriptome-metabolome-proteome association analysis results. Interaction network base on differentially expressed genes (DEGs), differential metabolites (DMs) and differentially expressed proteins (DEPs) that related to plant hormone signaling pathways. The circle in the network diagram represents DEPs, the triangle represents DEGs, and the downward angle represents DMs.

Further interactive network analysis was performed on core DEGs, DMs, and DEPs to identify key regulators that response to salt stress. The interactive network was generated using the co-expression information of DEGs, DMs, and DEPs based on the Pearson correlation from the transcriptome, metabolome, and proteome profile (**Fig 6**). The results showed that jasmonic acid had a co-expression relationship with multiple mRNAs and proteins. Two inositol-3-phosphate synthase proteins were at the core of the interaction network, and co-expressed with DELLA, HAI3.1, GID1B, Bnacnng59530D, BnaA05g01450D and BanA03g21360D. Meanwhile, we found that L-Cysteine interacted with multiple mRNAs, including CAT2 and other known transcription factors related to salt stress.

### qRT-PCR verification results

**Fig 7:**
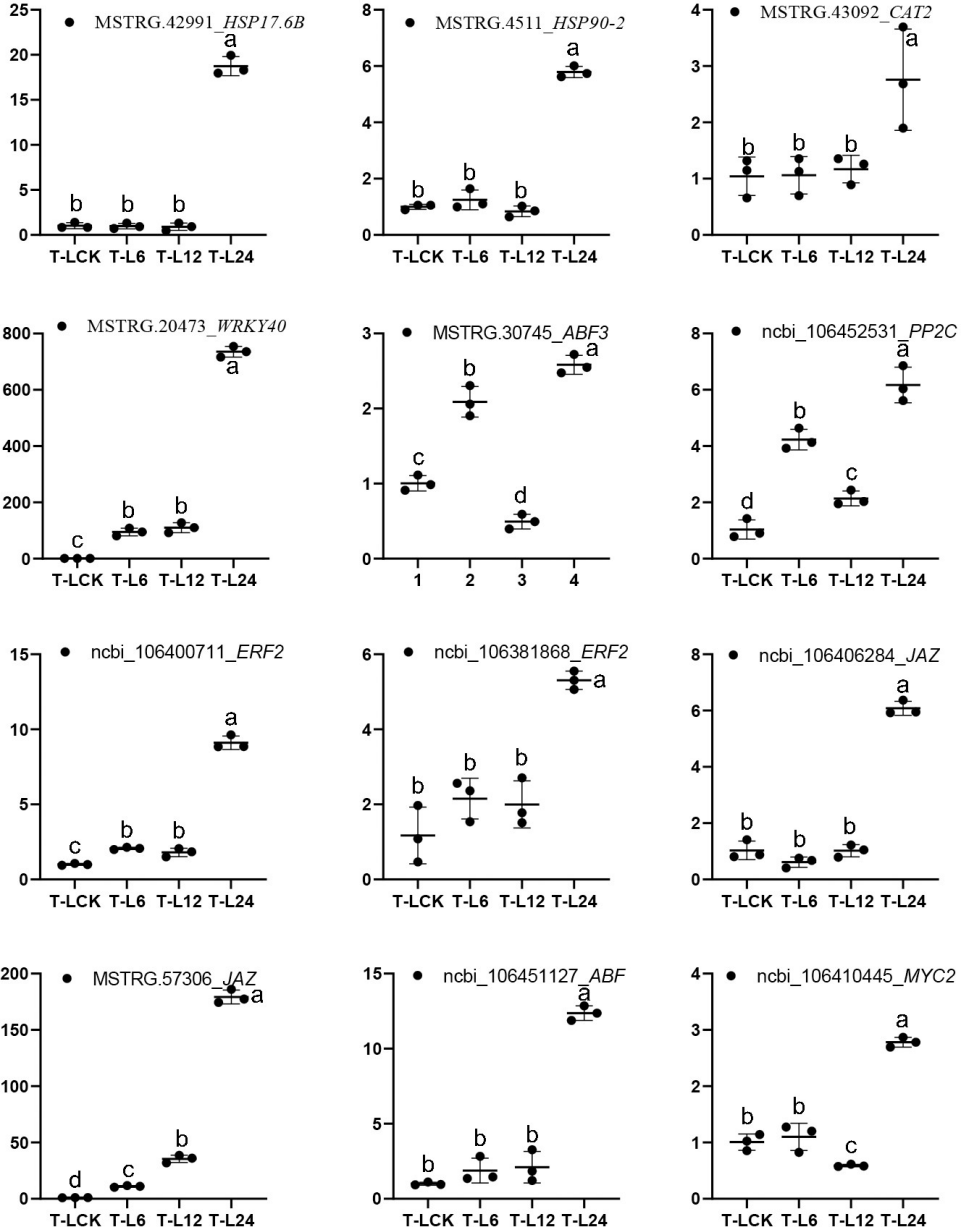
QRT-PCR analysis results. The range of gene expression was indicated by 2^−ΔΔCT^. Different letters represent significant differences (*p*< 0.05).

To validate the expression of candidate DEGs, we performed quantitative RT-PCR. The expression of *BnHSPs*, *BnCAT2*, *BnWRKY40*, *BnMYC*, *BnJAZ* etc. were significantly increased after 24 hours salt treatment (**Fig 7**). This result was similar to the variation tendency of signaling molecules observed in the above omics analysis, which indicated that the results observed in transcriptomics analysis were reliable.

## Discussion

Salt stress can induce toxicity and osmotic stress in plants. Our results also indicated that plant genes, metabolites, and proteomes had undergone great changes after salt stress. Plants use ABA-dependent and/or ABA-independent pathways to cope with abiotic stress [28]. The salt treatment activates sucrose nonfermenting 1‐related protein kinase 2 (SnRK2) kinase activity via increased ABA concentrations in plant cells [29–31]. PYR/PYL is a receptor of the ABA-signaling complex, and overexpression of PYR/PYL could suppress PP2Cs, which releases Snrk2s from the inhibition of PP2Cs, and subsequently activates the downstream target ABRE-binding factor [32]. While in the present study, all the *BnPP2Cs* were significantly increased after salt treatment, especially at 24h (Fig 3B). In contrast, all *BnPYR/PYLs* decreased. However, *BnABFs* showed a higher expression level at 24h after salt treatment. Plants struggling with stress need substantial energy to maintain their growth and development [33, 34]. Plants activate the ABA signaling pathway after being subjected to salt stress. When the *ABF* transcription factor is overexpressed, the up-regulated *PP2C* inhibits the ABA signaling pathway to save energy consumption. Moreover, many genes (such as *PP2C*) changed dramatically at 24h, indicating that 24h is a critical time point that *Brassica napus* began to inhibit the signal pathway transduction from saving energy consumption.

Other than that, the expression of *BnJAZs* was significantly increased after salt treatment, which prompted us to propose that jasmonic acid (JA) plays a key role in response to salt stress in *Brassica napus*. JAZ protein is a negative regulator in JA signaling, targeted by the E3-ubiquitin ligase SCF^COI1^ for 26S proteasome degradation in response to JA [35, 36]. When plants are subjected to environmental stresses, JAZ proteins have a reduced capacity to form complexes with COI1 in the presence of the receptor‐active form JA‐Ile and the potent agonist coronatine, which lead to the release of transcription factors like *MYC2*, etc. [37, 38]. When JA response occurs, *JAZs* are also induced, and then the expression of *MYC2* is consistent again to avoid the excessive consumption of plant energy [39, 40]. The dramatically increased *JAZs* at 24h after salt treatment indicated that plants have begun to suppress the JA signaling pathway to prevent plants from excessive energy consumption. A previous study reported that Jasmonate zim-domain protein 1/4 (JAZ1/4) proteins, which are repressors of the JA signaling pathway, interact with ICE1/2 to regulate CBF expression [41], suggesting that plants integrate hormone and cold signaling pathways for better adaptation to cold stress. In the present study, all the transcripts of *BnJAZ* were up-regulated by salt stress, especially peaked at 24h. The interactive network analysis of DEGs, DMs and DEPs in “plant hormone signaling pathways” showed that *BnJAR1_4_6* also showed the same expression trend with *BnJAZ* (**Fig 7**).

Interestingly, sn-glycero-3-phosphocholine connects the two sub-networks where *BnJAR1_4_6* and *BnPP2C* were located. Sn-glycero-3-phosphocholine, catalyzed by choline kinase (CK), is an intermediate in the synthesis of phosphatidylcholine (PC) [42]. Phosphatidylcholine (PC), phosphatidylethanolamine (PE), phosphatidylinositol (PI), and phosphatidylglycerol (PG), play different roles in plants’ response to salt stress [43]. The increased sn-glycero-3-phosphocholine indicated the increase of PC. Maintaining a higher level of PC is helpful for plant salt tolerance, which has been verified in Yu’s research on potatoes [44]. *BnJAR1_4_6* and *BnPP2C* are the key genes in the JA and ABA signaling pathways, respectively. In our results, the expressions of *BnJAR1_4_6* and *BnPP2C* are synergistic, indicating that JA and ABA are closely related in response to salt stress. ABA, Salicylic acid (SA), and JA are well known to have combined effects in plant development and plant defense [45]. Srivastava et al concluded that ABA level was regulated temporally which facilitated the induction of jasmonate to prioritize defense over growth in NaCl & Thiourea treatment in *Brassica juncea* [46]. Based on the results of this study, we speculate that both ABA, JA and SA play a synergistic role in response to salt stress in *Brassica napus*. Some cytochrome P450 (CYP450) family members, like allene oxide synthase (AOS), are potential catalysts for JA biosynthesis [47]. In our results, the CYPs proteins were accumulated after salt treatment, which might be related to the synthesis of JAs, and more works are needed to verify it. The high expression levels of CYPs showed that they could play a role in plants under drought stress conditions. Plant CYPs consist of a wide variety of isoforms with varied functions and cellular location. They are deemed to play crucial roles during growth and development, protein maturation and trafficking, besides processing of the nucleic acids [54, 55]. The increased CYPs showed the response to salt stress. Correspondingly, the mRNA of *BnCYPs* is also differently expressed, which might be the leading cause of changes in the CYPs protein level.

Moreover, the changing trend of betaine level was prominent in all DMs. Glycine betaine (GB) can protect plants from abiotic stresses via adjusting cellular osmotica and protecting membrane integrity [48, 49]. It has been reported that the accumulation of GB in plant tissues is correlated with higher salt stress tolerance in plants [50–53]. In the early stages of salt stress (12 hours before), GB level decreased, but by 24 hours, GB had a higher accumulation. This may be due to that in the early stage of salt stress, the GB present in plant cells was used to resist ROS, and after 12 hours, their own GBs could not continue to resist a large number of ROS, and then the cells synthesized a large number of GB in response to salt stress. More experiments are necessary to verify the conjecture. In addition, N-Acetylserotonin (NAS) decreased during the early stages of salt stress. NAS is an intermediate of serotonin metabolism and a precursor in melatonin synthesis [54, 55]. We speculate that the decreased NAS was used for melatonin synthesis, further used for ROS removal. N-Acetyl-5-hydroxytryptamine is the precursor of N-acetyl-5-methoxytryptamine, a compound that directly scavenges the ROS induced by various stress conditions [56, 57]. Therefore, the down-regulation of N-acetyl-5-hydroxytryptamine suggests that salt treatment triggered the overproduction of ROS in leaves. In summary, the down-regulation of N-acetyl-5-hydroxytryptamine appear to be part of a systematic response strategy employed by *Brassica napus* against salt-induced oxidative stress. Cysteine is an α-amino acid with a thiol side chain which participates in enzymatic reactions. L-cysteine act as a precursor for essential biomolecules, such as glutathione, vitamins and some other defense compounds [58, 59]. The increased L-cysteine indicated that plants are well prepared to accumulate substances that resist salt stress. The close relationship between L-cysteine and *Brassica napus* salt tolerance showed that L-cysteine might be used as a molecular marker. In addition, we found that HSP90s were highly expressed after salt treatment. The role of HSP90 proteins in plants in response to salt stress has been reviewed [60–62]. These HSP90 proteins changes would make plants less oxidatively damaged under salt stress, thereby conferring tolerance to salt stress. The accumulation of GB and HSP90 play important roles in the resistance of rapeseed to salt stress.

## Conclusion

Under salt stress, the jasmonic acid pathway responded positively in *Brassica napus*. The abundance of mRNA *BnJAZ* and DMs Jasmonic acid were increased by salt stress and they peaked at 24 hours of salt stress. Proteins like HSP, CAT, CYP were also activated and expressed by salt stress. In addition, some metabolites, such as N-acetyl-5-hydroxytryptamine, L-Cysteine and L-(+)-Arginine, play a critical role in maintaining the balance of ROS. The changes of these critical regulators indicated that the response of *Brassica napus* to salt stress is multifaceted, and the jasmonic acid signal might play a more important role in the process of salt stress resistance.

## Supporting information

S1 Table Primer sequence. (XLSX)

S2 Table Sequence data summary. (XLSX)

S1 Fig Basic information of sequencing data. (a). Principal component analysis (PCA) based on gene expression. (b). Samples correlation heatmap. White coloring indicates no correlation, while progressively darker green coloring indicates proportionally stronger correlations. (c). Number of DEGs in each comparison. Red and cyan bar indicate up- and down-regulated expression, respectively. (d). Venn analysis results of different comparisons.
(TIF)

S2 Fig Basic information of LC-MS data. (a). Principal component analysis (PCA) based on metabolite abundance. (b) to (e) shows the PLS-DA scores clustering results of CK vs. T3, CK vs. T6, CK vs. T12, and CK vs. T24, respectively.
(TIF)
