# Supplementary figures and images for: Transcriptomic, proteomic, metabolomic, and functional genomic approaches of Brassica napus L. during salt stress
Source: PLoS One. 2022 Mar 10;17(3):e0262587. doi: 10.1371/journal.pone.0262587 (PMC8912142; doi:10.1371/journal.pone.0262587)

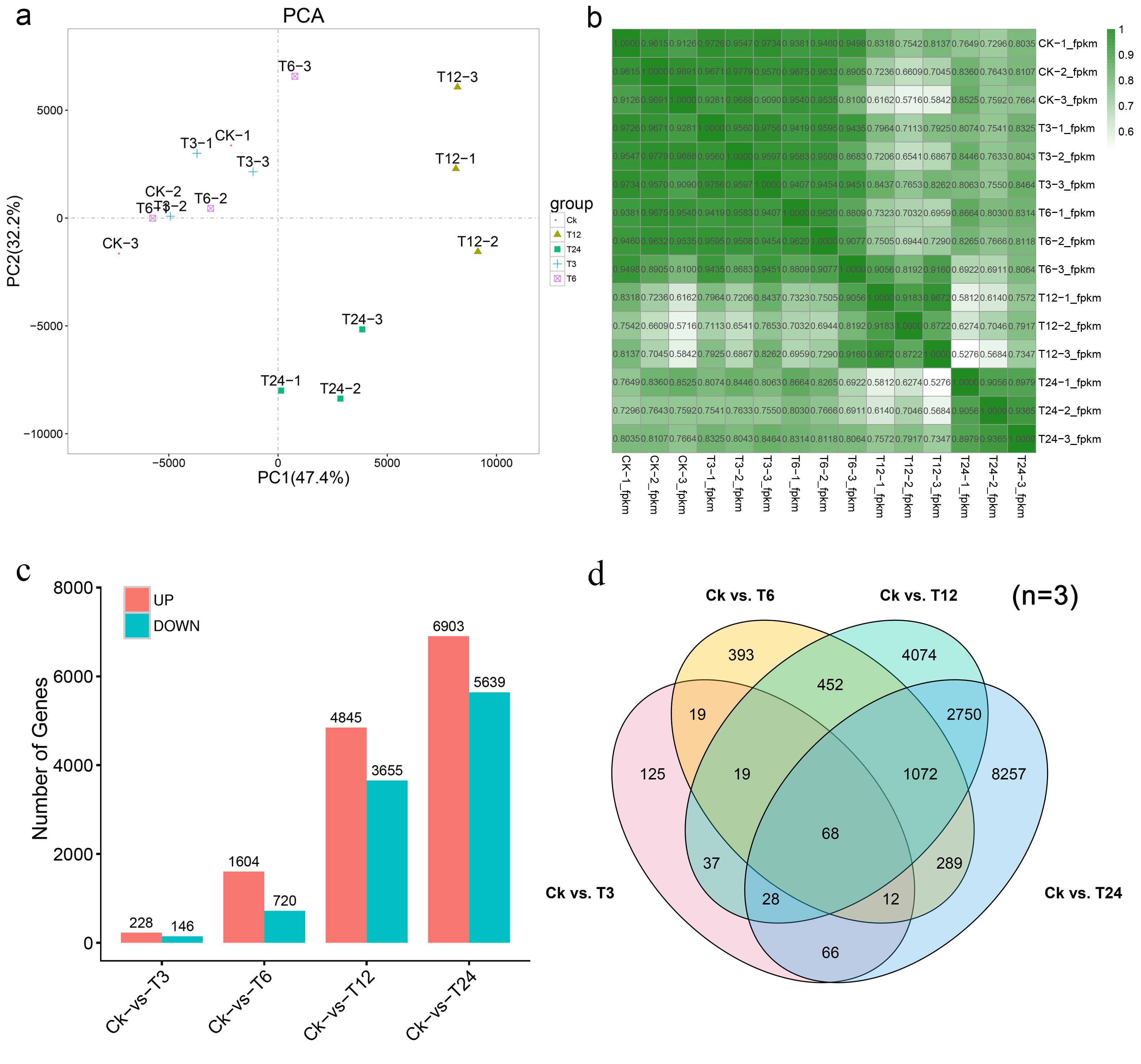

Supplement: S1 Fig — (a). Principal component analysis (PCA) based on gene expression. (b). Samples correlation heatmap. White coloring indicates no correlation, while progressively darker green coloring indicates proportionally stronger correlations. (c). Number of DEGs in each comparison. Red and cyan bar indicate up- and down-regulated expression, respectively. (d). Venn analysis results of different comparisons. (TIF) [file pone.0262587.s003.tif]

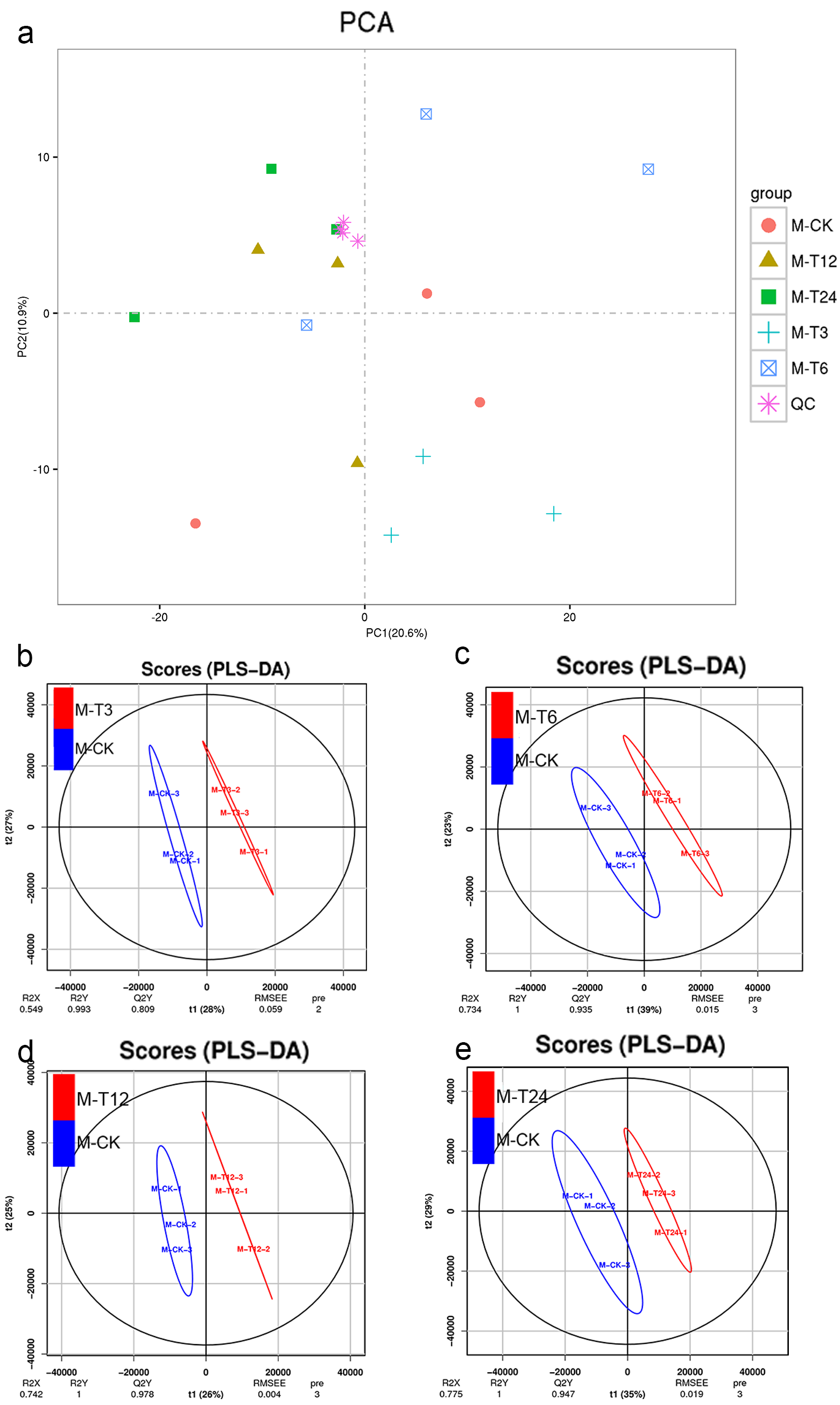

Supplement: S2 Fig — (a). Principal component analysis (PCA) based on metabolite abundance. (b) to (e) shows the PLS-DA scores clustering results of CK vs. T3, CK vs. T6, CK vs. T12, and CK vs. T24, respectively. (TIF) [file pone.0262587.s004.tif]
